# Acute myopathy secondary to oral steroid therapy in a 49-year-old man: a case report

**DOI:** 10.1186/1752-1947-5-82

**Published:** 2011-02-25

**Authors:** Muhammad A Khan, Eric Larson

**Affiliations:** 1 Department of Internal Medicine, Sanford School of Medicine, Health Science Center, 1400 West 22nd Street, Sioux Falls, SD 57105, USA

## Abstract

**Introduction:**

Acute myopathy caused by oral corticosteroids is rare. We present a case of myopathy occurring after two doses of methylprednisolone. Typically, acute steroid myopathy occurs with therapy using intravenous corticosteroids at high doses. Acute myopathy developing very early in the course of treatment with oral corticosteroids has been reported only once in the literature. Corticosteroid therapy may be complicated by myopathy, usually chronic, after prolonged high-dose therapy. Acute myopathy caused by exogenous corticosteroids is rare, usually with intravenous corticosteroids at high doses.

**Case presentation:**

A 49-year-old Caucasian man developed acute myopathy after taking oral methylprednisolone for only two days, 24 mg on day 1 and 20 mg on day 2. He discontinued the medication because of new-onset myalgias and lethargy on day 3 and was seen in our clinic four days after beginning therapy. He completely recovered in four weeks by discontinuing the corticosteroids.

**Conclusion:**

Among the many complications of corticosteroid therapy, acute myopathy is very rare. It requires prompt recognition and adjustment of therapy.

## Introduction

In 1932, Cushing [1] described myopathy as a clinical feature of hypercortisolism. Corticosteroids were introduced into clinical practice in 1948, and in 1958, Dubois [2] reported the first patient with myopathy resulting from iatrogenic corticosteroids. Since corticosteroid therapy's introduction into clinical practice, both acute and chronic steroid myopathies have been well recognized.

Chronic steroid myopathy is more common and develops after prolonged usage of steroids [3,4]. Acute steroid myopathy (ASM) is less common and develops early in the course of treatment, typically with high-dose intravenous (IV) steroids [4].

Earlier case reports of ASM usually involved patients with asthma receiving high-dose IV corticosteroids for status asthmaticus [5,6]. MacFarlane and Rosenthal [5] reported a case of a patient receiving IV hydrocortisone and developing myopathy manifested as difficulty weaning from a ventilator. Acute myopathy developing from oral corticosteroid has not been often reported. Kumar [7] described a patient developing myopathy after one dose of oral corticosteroid therapy. We report a similar case in which the patient developed acute myopathy after two doses of methylprednisolone.

## Case presentation

A 49-year-old Caucasian man presented to the local orthopaedic clinic with complaints of pain in the sole of his foot. He was diagnosed with plantar fascitis and was given a prescription for a methylprednisolone dose pack. He complained of vague neck pain on day 2 of therapy. Initially, he ignored the symptoms, but his condition continued to escalate in a generalized fashion. On day 3 of therapy, myalgia and muscle weakness progressed to involve upper arm and thigh muscles, and he did not take the methylprednisolone dose. He was seen on day 4 of therapy by which time his symptoms had progressed to generalized muscle weakness and pain. He stated that he was unable to open his car door because of hand weakness. He also complained of myalgias involving the same muscle groups. He was tender to palpation, and his symptoms did not improve with 500 mg of over-the-counter acetaminophen, which he was taking every six hours. He had no fever, difficulty breathing, flu like symptoms, facial muscle weakness, difficulty swallowing, or urinary or gastrointestinal symptoms. Before the current illness, his medical history was significant for gastroesophageal reflux disease.

On examination, his vital signs included blood pressure of 130/85 mm Hg, pulse of 80 beats/min, respiratory rate of 15 breaths/min, temperature of 98.9°F and oxygen saturation of 98% on room air by pulse oximetry. There was pain on palpation of the upper and lower extremity musculature, including the small muscles of the hand. Sensation in cranial and peripheral nerve distribution was normal and symmetric bilaterally. Muscle strength was 2 of 5 in the flexor and extensor groups of the upper and lower extremities. Facial muscle strength was normal. His hand grip was weak, and he had difficulty standing up from a sitting position. His gait was normal.

Normal deep tendon reflexes were noted. Babinski's sign was absent. He had normal cardiovascular, respiratory and abdominal examination results.

On laboratory examination, the patient had levels as follows: creatinine phosphokinase (CPK) of 891 U/L (reference range, 22-198 U/L), alkaline phosphatase (ALP) of 77 IU/L (reference range, 30-120 IU/L), aspartate aminotransferase (AST) of 64 U/L (reference range, 10-40 IU/L), alanine aminotransferase (ALT) of 69 IU/L (reference range, 9-60 IU/L, C-reactive protein (CRP) of 14.86 mg/L (reference range, <5 mg/L), and erythrocyte sedimentation rate (ESR) of 10 mm/hr (reference range, Age/2). Muscle biopsy and electromyography (EMG) were not performed. A basic metabolic panel and complete blood count results were normal.

He was sent home with ibuprofen 400 mg every six hours as needed for myalgias and instructions to call if his symptoms worsened. He was seen at a one-week scheduled return visit and reported significant improvement in muscle strength and decreased pain. Examination showed muscle strength of 5 of 5 in all muscle groups. Laboratory examination showed a CPK of 130 U/L, ALT of 82 IU/L and AST of 44 U/L. Urine myoglobin results were negative.

He was seen again 30 days after initial presentation and was feeling fine and had resumed his normal active lifestyle, farming. He continues to complain of intermittent foot pain, which is worse with activity, and he takes ibuprofen 400 mg as needed for relief.

## Discussion

ASM is rare and poorly understood. Several theories have been proposed to explain the pathogenesis of this condition. One model proposes activation of ubiquitin-dependent proteolytic systems [8]. Another model suggests that insulin-like growth factor-1, which may act as anti-apoptotic, is inhibited by steroids, thus allowing increased muscle apoptosis [9].

Askari *et al. *[10] recognized ASM in six of eight patients who were receiving oral prednisone therapy between July 1972 and November 1973. One patient developed symptoms of ASM within a few days of starting therapy. Five patients tolerated a low maintenance dose of prednisone (15 to 60 mg) for a duration of 60 to 240 days without any signs or symptoms of myopathy. However, increasing the maintenance dose resulted in the appearance of symptoms of corticosteroid myopathy within 30 days in four of five patients. The group concluded that the development of myopathy in patients receiving corticosteroid therapy is not related to the age of the patient, the magnitude of the dose given or the duration of maintenance therapy [10].

Typical presentations include diffuse myalgias and muscle weakness. Pelvic girdle muscle involvement is most consistently seen [10]. Some patients present with difficulty weaning from mechanical ventilators [5,6]. Our patient received methylprednisolone, and he was not on any maintenance corticosteroid therapy. He did have pelvic girdle muscle weakness, but his symptoms were not limited to these muscle groups.

A number of laboratory investigations may aid in the diagnosis of ASM. These include serum markers such as CPK, AST and ALT and urine markers, including urine myoglobin. EMG and muscle biopsy may also be helpful. No single test is diagnostic for this condition. The diagnosis always involves a high degree of clinical suspicion with diagnostic tests as an adjunct.

Serum marker elevation is an inconsistent finding. Our patient, including other reported cases [6,7], had elevated CPK, AST and ALT. Askari *et al. *[10] did not find CPK elevation as a consistent finding in their reported cases. They did find increased urinary creatinine excretion to be more consistently abnormal than elevated CPK [10].

EMG may be normal, but abnormal EMG findings classically include normal sensory and motor conduction velocities with decreased amplitude of muscle action potential [11]. We did not obtain EMG in our patient. Typically, muscle biopsy shows diffuse necrosis of both type I and type II fibers [6,11]; however, muscle biopsy is often diagnostically unhelpful [10].

Currently, there are no dosing recommendations for steroids that could decrease the likelihood of developing myopathy. Our patient received two doses of methylprednisolone, 24 mg and 20 mg. The only similar report involved a patient taking 40 mg of prednisone [7]. We were unable to find literature reports of patients developing myopathy while taking doses less than 40 mg of prednisone.

There is no specific treatment available for this condition. The most consistent finding on literature review is the fact that myopathy resolves without any intervention with discontinuation of steroid therapy.

## Conclusion

Steroids, as a class, are well recognized as a critical treatment modality for a number of conditions. They are prescribed by physicians belonging to almost all areas of medicine. Although very rare, ASM needs to be recognized early to ameliorate its significant effects.

## Abbreviations

ALP: alkaline phosphatase; ALT: alanine aminotransferase; ASM: acute steroid myopathy; AST: aspartate aminotransferase; CPK: creatinine phosphokinase; CRP: C-reactive protein; EMG: electromyography; ESR: erythrocyte sedimentation rate; IV: intravenous.

## Consent

Written informed consent was obtained from the patient for publication of this case report. A copy of the written consent is available for review by the Editor-in-Chief of this journal.

## Competing interests

The authors declare that they have no competing interests.

## Authors' contributions

MAK wrote the case report and searched the literature. EL is the primary care physician of the patient. EL carried out the final revision of the manuscript. Both are involved in patient management. Both authors read and approved the final manuscript.
